# Two rare cases of primary clear cell adenocarcinoma of the urethra: clinical experience, case report and literature review

**DOI:** 10.3389/fonc.2025.1539312

**Published:** 2025-02-12

**Authors:** Bohao Jiang, Jiyuan Hu, Benqiao Wang, Xujia Liu, Ling Tong, Yitong Xu, Hao Zhang

**Affiliations:** ^1^ Department of Urology, The First Hospital of China Medical University, Shenyang, Liaoning, China; ^2^ Department of Neurology, The First Hospital of China Medical University, Shenyang, Liaoning, China; ^3^ Department of Rehabilitation, Shengjing Hospital of China Medical University, Shenyang, Liaoning, China; ^4^ Department of Surgery, The First Hospital of China Medical University, Shenyang, Liaoning, China; ^5^ Department of Pathology, The First Hospital of China Medical University, Shenyang, Liaoning, China

**Keywords:** urethral carcinoma, primary clear cell adenocarcinoma, case report, literature review, surgical treatment

## Abstract

**Background:**

Primary clear cell adenocarcinoma of the urethra (CCAU) is a kind of extremely rare genitourinary cancer. Despite the similarity in the clinical manifestations of these reported cases, diagnosis and determination of standard therapy remain challenging due to the rarity of findings and similarity with other urethral tumors.

**Case presentation:**

Herein, we reported two cases of CCAU with the same chief complaint of hematuria: a 71-year-old female and a 66-year-old male. The male patient reported concomitant symptoms of frequent and painful urination. CT scans show abnormal enhancements. After a cystoscopy examination, both patients are diagnosed with malignant urethral tumors. Surgical resections and additional pathological examinations support the diagnosis of CCAU (palliative resection for case 1 and transurethral resection for case 2). Case 1 undergone progression 6 months after initial treatment with transurethral resection and chemotherapy with a 15-month overall survival. In contrast, the prognosis of case 2 remained uneventful 10 months after surgery without recurrence. After presenting our cases, we launched a literature review that included 23 articles and 33 cases of CCAU to summarize the characteristics of the disease.

**Conclusion:**

Primary clear cell adenocarcinoma of the urethra is a rare malignant urethral tumor with controversial histological origins. Primary symptoms include hematuria and changes in voiding habits. Middle-aged and elderly females are more susceptible to primary clear-cell adenocarcinoma of the urethra. Unfortunately, it is difficult to differentiate primary clear-cell adenocarcinoma of the urethra from other urethral tumors due to similar clinical features. However, imaging tools such as CT, MRI, and cystoscopy are adjunctive in confirming diagnoses. Even though surgical resection is the primary treatment to relieve clinical symptoms, prevent recurrence, and confirm diagnosis, no standard surgical protocol is available. The therapeutic effect of postoperative adjuvant therapies remains unclear. Future investigations on CCAU are necessary to advance clinical knowledge and to provide treatment guidance.

## Introduction

Primary clear cell adenocarcinoma of the lower urinary tract is a rare type of malignant genitourinary tumor that can be found in both the bladder and urethra. Less than 100 cases are reported in English, leading to difficulty in diagnosis and treatment. The urethra is even a rarer tumor location than the bladder, with only dozens of cases reported. Due to the rarity of this type of tumor, there needs to be a better understanding of the tumor’s growing behavior, clinical manifestations, and treatment strategies.

Herein, we report two cases of clear cell adenocarcinoma of the urethra (CCAU) undergoing surgical treatment: a 71-year-old female patient and a 66-year-old male patient. We start with a detailed description of patients’ main symptoms, radiological presentions, therapeutic experiences, pathologic features, and prognosis, which was displayed in **Table 1**. We then launched a literature review on clear cell adenocarcinoma of the urethra to provide a conclusion on the reported cases that includes clinical features and treatment modalities.

**Table 1:** Basic characteristics of our 2 reported cases.

Patient ID	Case 1	Case 2
Age	71	66
Gender	F	M
Symptoms	hematuria & interrupt urination	hematuria, frequent and painful urination
Size (cm)	2.7*2.2	1.4*0.9
Primary treatment	Transurethral resection and chemotherapy	Transurethral resection
Subsequent treatment	Palliative urethrectomy and cystostomy due to progression	NA
Postoperative hospitalization	4 days	8 days
Follow-up	Local recurrence 6 months after palliative surgery; Multiple distal metastasis 7 months after palliative surgery	No recurrence
Progression-free Survival	6 months	Not reached
Overall survival	15 months	Not reached
IHC Results	CK(+), Villin(+), SATB2(-), CK7(+), CK20(-), P53(wild type), P63(-), Ki-67(60%+), GATA-3(-), C-erbB-2(2+), CDX-2(-), ER(-), PR(-), P16(-), P16(-), WT1(-), PAX8(+), Napsin-A(-), HNF1β(+), NKX3.1(-), PSA(-)	CK7(+), CK20(partial +), GATA-3(partial weak +), Ki-67(70%+), HNF1β(+), Napsin-A(-), PAX8(+), P53(-), P63(-), PD-L1(clone SP263)(TC=5%, ICP=10%, IC+=80%).

IHC, Immunohistochemistry.

## Case 1

**Figure 1:**
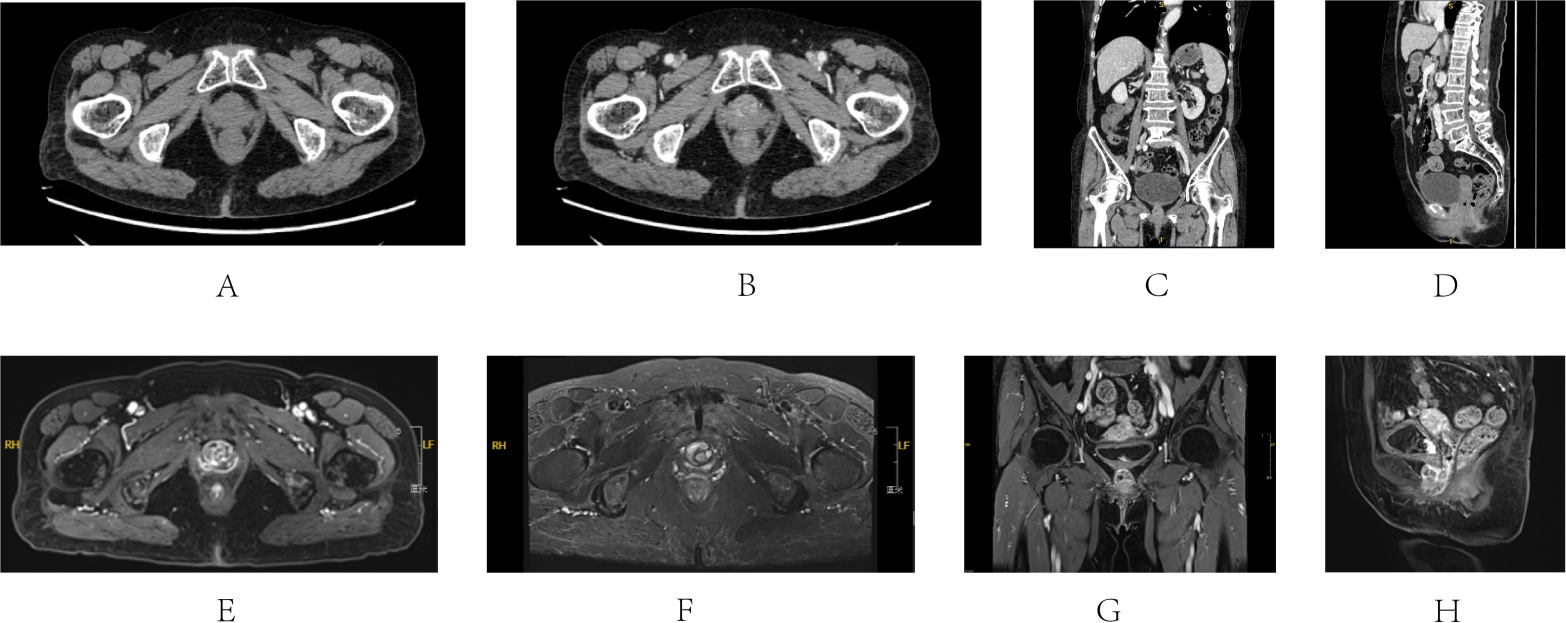
CT and MRI images of Case 1’s first visit to the hospital (March 2022). CT **(A-D)** A 2.5*2 cm nodule was found on the urethra, with a plain CT value of 45 HU, and enhancement was visible. MRI **(E-H)** A 2.7*2.2 cm cystic signal was seen with long signal at both T1 and T2 stage, and significant enhancement of the margins and cystic septa can be seen. **(A)** Axis plain CT scan of the mass; **(B)** Axis enhanced CT scan; **(C)** Coronal enhanced CT scan; **(D)** Sagittal enhanced CT scan; **(E)** Axis T1 stage of enhanced MRI; **(F)** Axis T2 stage of enhanced MRI; **(G)** Coronal T1 stage of enhanced MRI; **(H)** Sagittal T1 stage of enhanced MRI.

A 71-year-old female patient presented to our outpatient with a primary complaint of persistent, painless hematuria and interrupted urination for 8 years in March 2022. She denied any other symptoms, such as urinary frequency and urgency. The patient has a past medical history of hypertension that has been well-controlled with oral nifedipine for the past five years and diabetes mellitus without medication management in the past year. Physical examinations showed no abnormalities. The patient received a CT and MRI examination in the outpatient clinic (**Figure 1**). A 2.5*2 cm nodule was found on the urethra, with a plain CT value of 45 HU, and enhancement was visible in an enhanced scan (84 HU). The mass appeared as a 2.7*2.2 cm cystic signal, which was also seen on the MRI image (long signal at both T1 and T2 stage), and significant enhancement of the margins and cystic septa can be seen. The patient received further cystoscopy examination, in which a flat, pedunculated mass was seen 1 cm from the external urethral orifice. Considering the potential for malignancy, we suggested a biopsy and surgical excision, but the patient refused further treatments and chose conservative treatment instead.

One year later, the patient received a puncture biopsy of the mass at another facility due to worsened hematuria and urinary symptoms. Transurethral resection of the urethral tumor was performed in September 2023 after the pathology result returned as malignant. Due to the presence of pelvic and inguinal lymph node metastasis, chemotherapy (Cisplatin and Gemcitabine) was given after surgery. However, chemotherapy had to be stopped due to myelosuppression 3 months later. Significant response was observed from the CT images before and after chemotherapy but partial remission was not achieved (**Supplementary Figure 1**). In April 2024 (6 months after primary treatment of transurethral resection and chemotherapy), the patient returned to our hospital for sudden bleeding from the urethra. CT and MRI results indicated a recurrence of a urethral mass (2.2*1.8 cm in size), measuring 2.2 x 1.8 cm, along with progression of lymph node metastases in the left inguinal region and pelvic wall (**Supplementary Figures 2**, **3**). Cystoscopy showed a 2.5*1.5 cm cauliflower-like tumor located 0.5 cm from the external urethral orifice.

**Figure 2:**
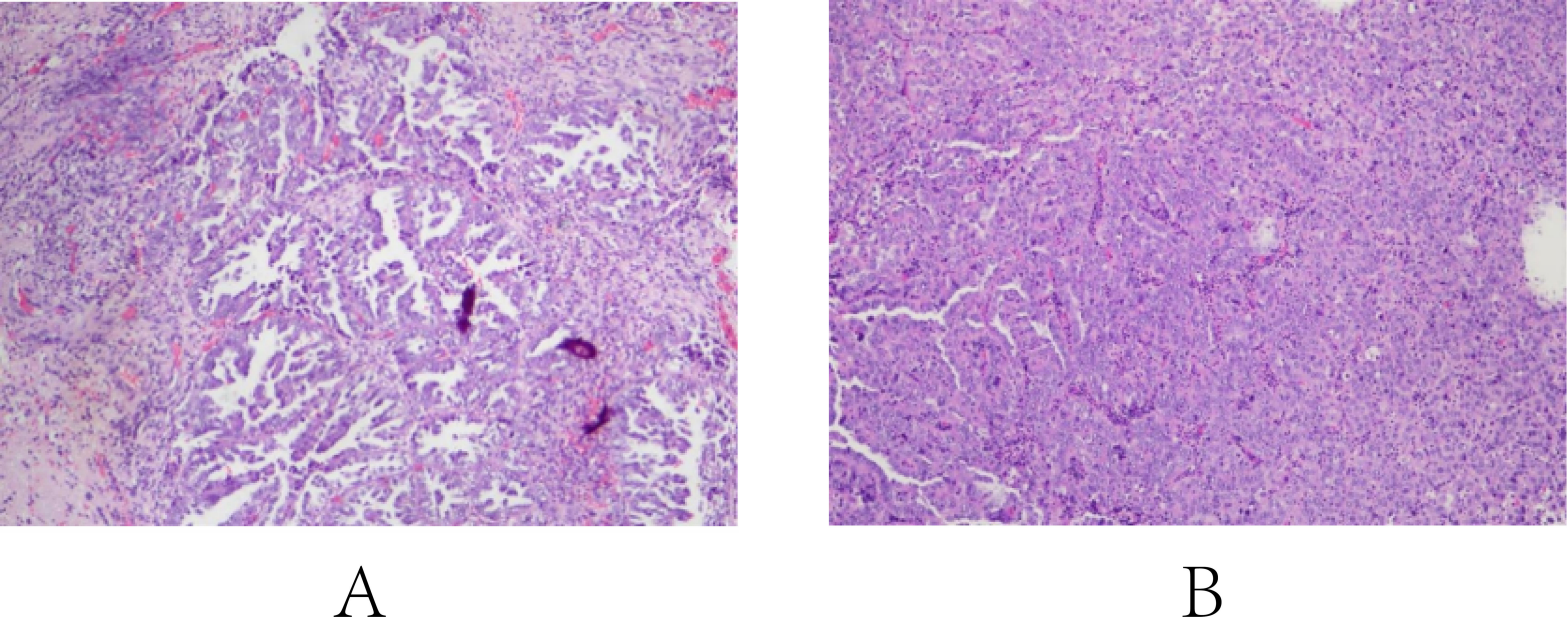
Pathological results of two cases: [**(A)** Case 1; **(B)** Case 2].

The patient underwent a palliative urethrectomy and cystostomy due to tumor recurrence. The entire length of the urethra, about 6.5cm, was removed. After dissecting along the long axis of the urethra, a cauliflower-like mass approximately 1.5*1.2 cm, with a grayish-white cut surface, was found invading the muscularis propria. The resected tissue was then sent to the pathology department. A final pathology diagnosis of T2NxM1 clear cell carcinoma was made with negative surgical margins (**Figure 2A**). Immunohistochemistry result was reported as follows: CK(+), Villin(+), SATB2(-), CK7(+), CK20(-), P53(wild type), P63(-), Ki-67(60%+), GATA-3(-), C-erbB-2(2+), CDX-2(-), ER(-), PR(-), P16(-), P16(-), WT1(-), PAX8(+), Napsin-A(-), HNF1β(+), NKX3.1(-), PSA(-). The patient declined adjuvant chemotherapy and immunotherapy and chose active surveillance instead. The patient was discharged on the fourth day after surgery and received regular postoperative follow-up. 6 months after surgery (12 months after primary treatment), the tumor locally recurred, presenting as a spherical mass growing outward from the bladder fistula site. Multiple distal metastases of the liver portal lymph nodes, retroperitoneal lymph nodes, and bilateral inguinal lymph nodes were identified a month later. Additionally, metastatic tumor was found in the right adrenal gland area (**Supplementary Figures 4**, **5**). The patient refused any further treatment except for bladder fistula tube replacement and pain management. She passed away in January 2025, with an overall survival of 15 months since the diagnosis of CCAU (9 months after palliative surgery).

## Case 2

**Figure 3:**
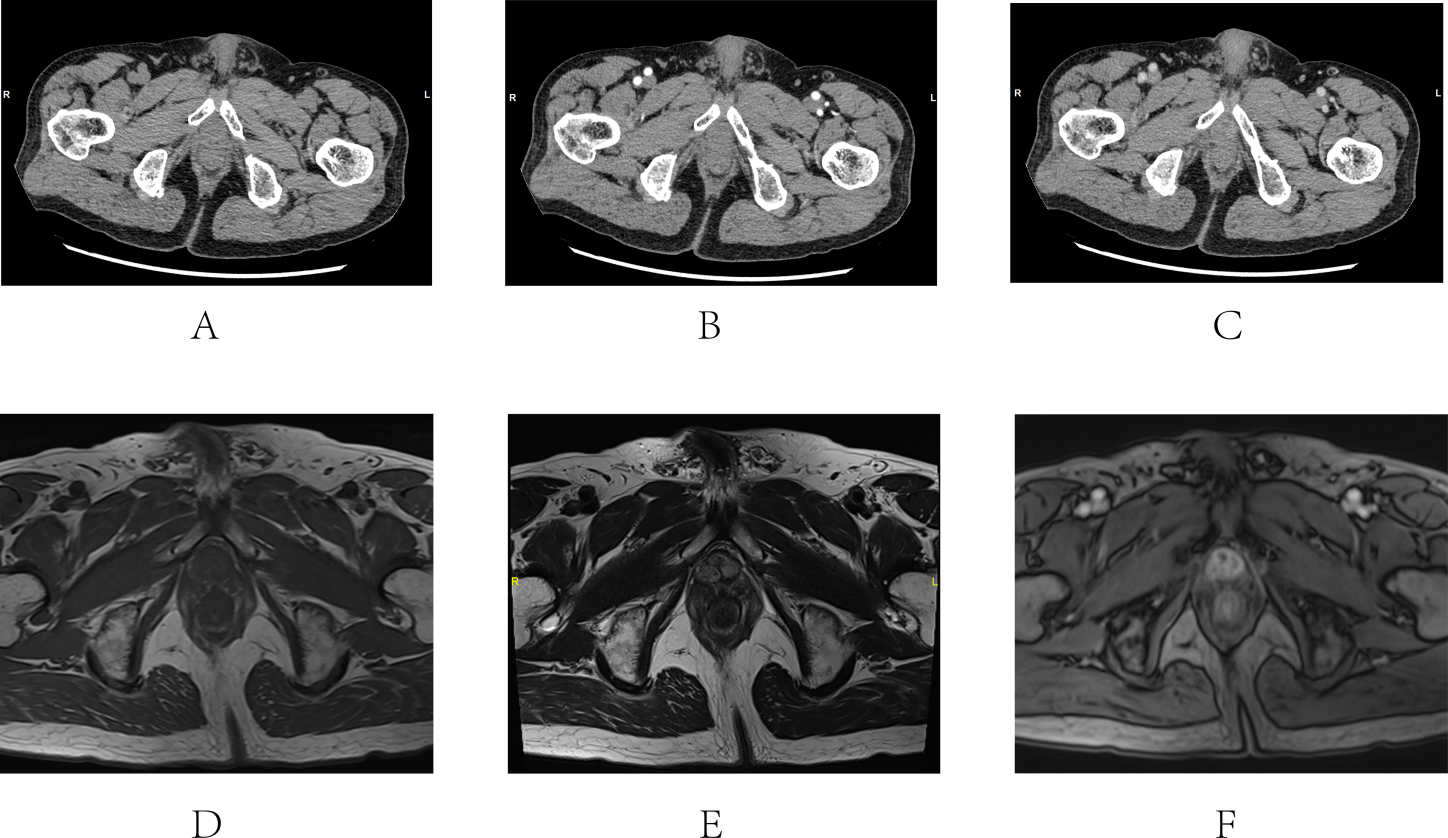
CT and MRI images of Case 2. CT **(A-C)** An uneven thickening of the prostatic urethra near the bladder neck with a slight enhancement. MRI **(D-F)** 1.4* 0.9cm nodule was seen at the urethral orifice with a long T2 signal and circular enhancement. **(A)** Plain scan of the mass; **(B)** Enhanced scan arterial phase; **(C)** Enhanced scan venous phase; **(D)** T1 stage of MRI; **(E)** T2 stage of MRI; **(F)** Enhanced T1 stage of MRI.

A 66-year-old man came to our hospital complaining of hematuria with occasional blood clots in urine for six months, which has aggravated two months ago. The patient also reports urinary frequency and dysuria. The patient has a past medical history of hypertension for five years and is well-controlled with oral enalapril. He also has a past surgical history of transurethral resection of the prostate (TURP) for prostatic hyperplasia two years ago, with a pathology diagnosis of clear cell carcinoma. Given the unusual pathology, the patient underwent cystoscopy to rule out other histological sources. No obvious abnormality was found except for patchy mucosal redness near the bladder neck. Regular surveillance was maintained after the TURP surgery. The patient’s physical examination was regular. There were no abnormal laboratory values except for the routine urine test, in which the white blood cell counts were slightly elevated, indicating a potential urinary tract infection. Infection is controlled with intravenous levofloxacin (0.5g, qd) for six days. CT and MRI examinations were conducted for radiological diagnosis (**Figure 3**; **Supplementary Figure 6**). On the CT image, an uneven thickening of the prostatic urethra near the bladder neck was seen with a slight enhancement at the enhanced phase. Multiple nodules could be seen at the urethral orifice of the bladder on MRI images with a long T2 signal and circular enhancement, the largest of which was 1.4*0.9 cm. Cystoscopy found a papillary tumor in the prostatic urethra, during which the pathology sampling was completed simultaneously, suggesting a pathology diagnosis of primary clear cell adenocarcinoma of the urinary tract.

After the urinary tract infection was treated, a transurethral resection surgery was performed to remove the tumor and to confirm the pathology diagnosis. The pathology diagnosis was in line with the former results (**Figure 2B**). Further immunohistochemistry results were reported as following: CK7(+), CK20(partial +), GATA-3(partial weak +), Ki-67(70%+), HNF1β(+), Napsin-A(-), PAX8(+), P53(-), P63(-), PD-L1(clone SP263)(TC=5%, ICP=10%, IC+=80%). Considering the potential risk of local and distal recurrence, a further surgical protocol, including radical prostatectomy, total urethrectomy, and cystostomy, was recommended to the patient. After discussion and consideration, the patient refused to receive further surgery due to the poor quality of postoperative survival. Suggestions of regular active surveillance were also rejected. Regular follow-up phone calls revealed that the patient has an uneventful prognosis without recurrence of symptoms until January 2025 (10 months postoperatively).

## Discussion

Primary clear cell adenocarcinoma (CCA) is an extremely rare subtype of malignant tumor of the lower urinary tract. Swartz et al. reported that primary clear cell adenocarcinoma of the urethra (CCAU) accounts for 16.8% of urethral cancer, similar to the percentage of CCA of the bladder in bladder cancer (1–3). In addition to the rare incidence, it also has an atypical growth location, which is usually located in the urethral diverticulum and periurethral tissue, leading to difficulties in differential diagnosis not only between CCA and transitional-cell cancer but also urethral tumors and tumors of other organs nearby, such as bladder and vagina. Few reports on this disease result in an insufficient understanding of the etiology, diagnosis, and treatment. Several theories of the origin of CCAU have been proposed, such as the origin of paraurethral glands (Skene’s gland) or urothelial metaplasia, without a reached consensus (4, 5).

Some literature reviews have been published to summarize the disease’s clinical features, pathological manifestations, or genetic characteristics (6–9). However, drawbacks still exist because most reviews were limited to female patients. Articles in these reviews were also relatively incomplete due to the publication dates. To supplement previous reviews and thoroughly summarize the symptoms and characteristics of this disease, we launched a systemic review by searching articles on this disease written in English and available on Pubmed databases. As a result, 26 literature reports were published from 1973 to 2024, in which 33 cases of CCAU were reported. Detailed characteristics of reported cases are present in **Supplementary Table 1**.

Regarding demographic features, CCAU is susceptible to the middle-aged and elderly female population. According to our literature search, the ages of patients with a confirmed diagnosis of CCAU range from 36 to 71 years old, with a majority in their 60s and an average age of 55.5 (36-40: 4/33, 12.1%; 41-50: 6/33, 18.2%; 51-60: 12/33, 36.4%; 61-71:11/33, 33.3%). There is a significant difference in incidence between genders. Most reported cases happened in females (28/33, 84.5%) and only 5 male patients were diagnosed (5/33, 15.5%). In addition to the primary growing location of the urethra, 7 cases were reported to invade the trigone and neck of the bladder (10–16).

To understand the impact of CCAU on patients’ survival time, we conducted a preliminary survival analysis on 33 previously reported cases. The progression-free survival (PFS) and overall survival (OS) of each case were extracted and summarized from their respective reports, as shown in **Supplementary Table 1**. Overall, post-treatment progressions and deaths were observed in 33.3% and 30.4% of the reported CCAU patients respectively (8 progression events out of 24 cases/7 deaths out of 23 cases) with a median overall survival time of 40 months. We found a significant difference in PFS and OS between non-metastatic and metastatic patients preoperatively (P <0.01). Subgroup analysis based on the metastatic status was further conducted. The prognosis is generally better for cases without preoperative metastasis, with both median PFS and OS not reached. For preoperatively metastatic cases, the prognosis is relatively poor with a median PFS of 12 months and a median OS of 28 months. The Kaplan - Meier plots of these two survival endpoints were presented in **Supplementary Figure 7**. It should be noted that due to the short follow-up time in most reported cases and the rarity of the disease, the results of our survival analysis should be interpreted with caution, as the prognosis for non-metastatic cases may be overestimated.

No specific symptoms were identified that are unique to CCAU, whose symptoms are analogous to those observed in other types of urethral carcinoma (**Supplementary Table 1**). The main symptoms are hematuria (16/33, 48.5%), urinary habit changes (10/33, 30.3%), and urinary retention (8/33, 24.2%). Other symptoms were also reported in a small portion of patients, such as urinary tract infection (5/33, 15.2%), vaginal bleeding/menometrorrhagia (3/33, 9.1%), and pain (3/33, 9.1%). In addition, Whitworth et al. reported a case diagnosed incidentally during a routine physical examination without any symptoms (17). So, it is difficult to make a preliminary diagnosis only based on the symptoms due to the diversity. CT and MRI images can provide radiological evidence for its diagnosis and guide further treatment (18). It appears as a round or oval mass of soft-tissue density around the urethra on the CT image, with enhancement visible on the enhancement scan. Invasions may also be observed in some cases into surrounding tissues, such as the bladder and vagina. In a review of the MRI manifestation of CCA in the urethra, four differences were concluded between the characteristics of CCA and non-clear cell adenocarcinoma (NCCA) (18): 1) CCA is often associated with urethra diverticulum. 2) CCA has a lower height-to-width ratio compared with NCCA. 3) More frequent septation can be seen in the tumor. 4) More normal urethra could be preserved compared with NCCA. Due to the rarity of the disease, more cases should be reported to make a more comprehensive conclusion. In our two cases, the height-to-width ratios were slightly larger. According to the literature review, nearly half of the cases were reported without a urethra diverticulum. Tumors affecting the whole urethra were also reported due to their large size (19).

Cystoscopy was an option for a more precise diagnosis, in which papillary or flatted mass could be seen. However, the cystoscopy results could be normal in rare cases (20), highlighting the importance of pathological examination after either transurethral resection or puncture biopsy. In case of pathological features, CCAU is morphologically similar to clear cell carcinoma of the female reproductive tract. The 2004 edition of the World Health Organization Classification of urethral tumors classified it as glandular tumors, and the most recent 2022 editionclassifies it as Mullerian tumors under a new chapter. The primary CCAU often presents a papillary、 tubular cystic、solid hybrid structure with a clear、 eosinophilic、 boot-spike cell hybrid. In terms of immunohistochemistry, CK7 (11/14, 78.6%) and PAX (5/14, 35.7%) were commonly expressed. HNF-1β, NapsinA, CA125, and AMACR were also expressed, while urinary tract markers GATA-3 and P63 were rarely expressed (10, 20–24). Detailed immunohistochemistry features of 33 reported cases were presented in **Supplementary Table 1**. HNF-1β, expressed in CCAU with a much higher positive rate compared with urothelial carcinoma, is often overexpressed in ovarian clear cell adenocarcinoma. Thus this marker helps diagnose such disease but is not more helpful in determining the source (primary CCAU or female reproductive tract clear cell adenocarcinoma metastasis). The distinction between primary and secondary cases depends on clinical history and radiological evaluation, as they are not distinguishable at the immunohistochemical level.

Due to its high malignancy and invasiveness, surgical resection is the primary treatment strategy, which can remove the existing tumor, release the obstructive symptoms, and aid with pathological sampling. Still, there is no standard protocol for the surgery (25). Regardless of subtypes, local resection suits low-grade urethral malignant tumors (cT1-T2) (26). Some patients were reported to receive treatment of transurethral resection with a resectoscope (27). Case 2 in our report also only underwent transurethral resection, and he has had an uneventful prognosis until now. However, Dimarco et al. published a review on the prognosis of malignant urethral cancer after different surgical methods (25). In that article, partial urethrectomy was related to poor prognosis and high recurrence rate compared with radical urethrectomy. Thus, anterior pelvic exenteration for females and cystoprostatectomy for males are good options to reduce the recurrence risk and prolong the survival, especially for patients without detected metastasis, most of whom have a good prognosis. According to our literature review, nearly all the patients (26/32, 81.3%) received radical surgery as the primary treatment or after recurrences of partial resection, chemotherapy and radiological therapy. The necessity of lymph node dissection depends on the presence of lymph node metastasis.

For CCAU with lymphatic or distal metastasis, surgical resection of tumors and involved metastatic lymph nodes remains the treatment option for most patients (10/12, 83.3%). The therapeutic efficacy of radiotherapy and chemotherapy alone, used as the main treatment without surgery, is unreliable. As reported in two previous cases and our case 1, patients may exhibit poor response or be forced to discontinue medication due to serious adverse events, resulting in missed surgical opportunity (15, 28). Although the efficacy of radiotherapy and chemotherapy alone is uncertain, they were reported to be applied as potential supplementary treatments. Rane et al. reported a female patient who underwent two cycles of neoadjuvant chemotherapy, and no recurrence was observed during subsequent surveillance (12). Some cases were also treated with adjuvant chemotherapy and radiotherapy postoperatively, most of which experienced recurrence or died of CCAU within 1-3 years after surgery (10/14, 71.4%). It is worth noting that patients with unsatisfactory pathological results or radiological manifestations are more likely to receive adjuvant treatments, which results in a high mortality rate and an inaccurate reflection of therapeutic effect. Due to the limited number of reported cases related to chemotherapy and radiotherapy, as well as the lack of large-scale retrospective studies, the adjuvant role of radiotherapy and chemotherapy in treatments of CCAU needs further reporting and verification.

## Conclusion

Clear cell adenocarcinoma of the urethra is an extremely rare malignant tumor without specific symptoms and radiologic manifestations, which causes difficulty in the differential diagnosis with other types of urethral tumors. MRI and cystoscope may shed light on the diagnosis, which ultimately depends on the pathology. Considering the likelihood of high malignancy, surgery is the primary treatment option for most patients, while no standard surgical protocol has been proposed until now. The therapeutic effect of radiotherapy and chemotherapy remains to be determined due to insufficient supporting data from published case-control studies. Case reports and large-scale retrospective studies are required in the future to enhance the comprehension of this disease and facilitate the development of optimal treatment strategies.

## Data Availability

The original contributions presented in the study are included in the article/**Supplementary Material**. Further inquiries can be directed to the corresponding authors.

## Supplementary material

Supplementary Figure 1 Comparison of 3-month chemotherapy. (October to December 2023) efficacy of case 1. **(A)** CT image taken in September 2023 before chemotherapy. Thickened urethra and enlarged left inguinal lymph nodes were presented; **(B)** CT image taken in January 2024 after chemotherapy. The lesions in the urethra and left inguinal lymph nodes showed significant improvement.

Supplementary Figure 2 Axis scanning of CT and MRI images of Case 1 6 months after chemotherapy and before palliative surgery. CT **(A-C)** A 2.5^∗^1.9 cm nodule was found on the urethra, with a plain CT value of 40 HU, and enhancement was visible (70 HU); MRI **(D-F)** A 2.2*1.8 cm cystic signal was seen with long signal at both T1 and T2 stage with significant enhancement; Multiple enlargements of lymph nodes **(G-I)** in the left pelvic wall and the inguinal region presented on MRI images. [**(A)** Plain scan of the mass; **(B)** Enhanced scan arterial phase; **(C)** Enhanced scan venous phase; **(D)** T1 stage of MRI; **(E)** T2 stage of MRI; **(F)** Enhanced T1 stage of the mass; **(G–I)** Enhanced T1 stage of the enlarged lymph nodes].

Supplementary Figure 3 Coronal and sagittal images of MRI of Case 1 after chemotherapy recurrence, with a cystic urethral mass and enlarged left pelvic and inguinal lymph nodes. **(A–E)** Coronal sequences; **(F–J)** Sagittal sequences.

Supplementary Figure 4 Axis CT images of Case 1 (Progression and recurrence 6 months after palliative surgery): **(A)** Lymph node metastasis at the porta hepatis; **(B)** Metastatic tumor in the left adrenal area; **(C)** A mass growing outward from the bladder fistula site and left inguinal lymph node metastasis were presented; **(D)** Metastases of bilateral inguinal lymph nodes.

Supplementary Figure 5 Coronal and sagittal images of CT of recurrence after palliative surgery in case 1. **(A–C)** Coronal sequences; **(D–F)** Sagittal sequences. [(A) Enlarged and fused left inguinal lymph nodes; **(B)** Enlarged bilateral inguinal lymph nodes and left pelvic lymph nodes; **(C)** Metastatic right adrenal tumor; **(D)** Enlarged and fused left inguinal lymph nodes; **(E)** A mass growing outward from the bladder fistula site; **(F)** Metastatic right adrenal tumor].

Supplementary Figure 6 Coronal and sagittal images of MRI of Case 2 (with a nodule at the urethral orifice). **(A–D)** Represented a continuous sequence of coronal images. **(E–H)** Represented a continuous sequence of sagittal images.

Supplementary Figure 7 Kaplan-Meier Plots of progression-free survival (PFS) and overall survival (OS) of 33 previously reported cases. Consistent with clinical knowledge, patients with detected metastasis prior to treatment have shorter survival times and more common post-treatment progression. It is worth noting that the results of K-M plots can reflect the tendency of the difference in prognosis between these two groups, but may not be accurate enough due to the short follow-up time of most reported cases and the rarity of the disease. (Blue: patients without metastatic sites before primary treatments; Red: patients with detected metastatic sites.)
