# Supplementary figures and images for: Two rare cases of primary clear cell adenocarcinoma of the urethra: clinical experience, case report and literature review
Source: Front Oncol. 2025 Feb 12;15:1539312. doi: 10.3389/fonc.2025.1539312 (PMC11861585; doi:10.3389/fonc.2025.1539312)

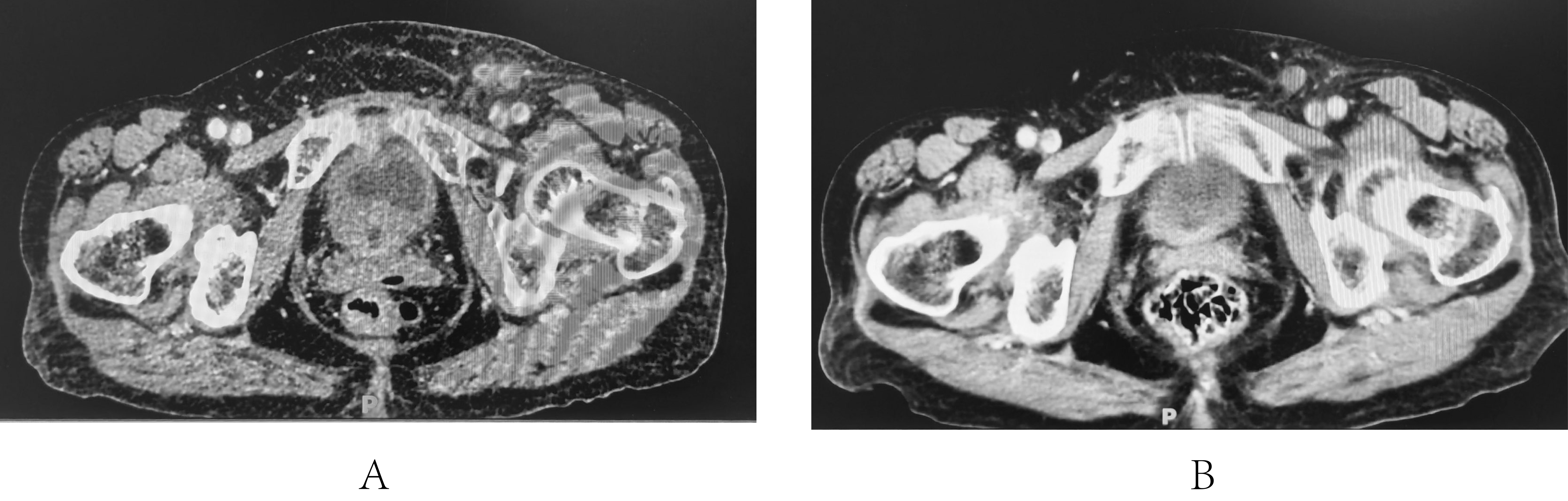

Supplement: Supplementary Figure 1 — Comparison of 3-month chemotherapy. (October to December 2023) efficacy of case 1. (A) CT image taken in September 2023 before chemotherapy. Thickened urethra and enlarged left inguinal lymph nodes were presented; (B) CT image taken in January 2024 after chemotherapy. The lesions in the urethra and left inguinal lymph nodes showed significant improvement. [file Image1.jpeg]

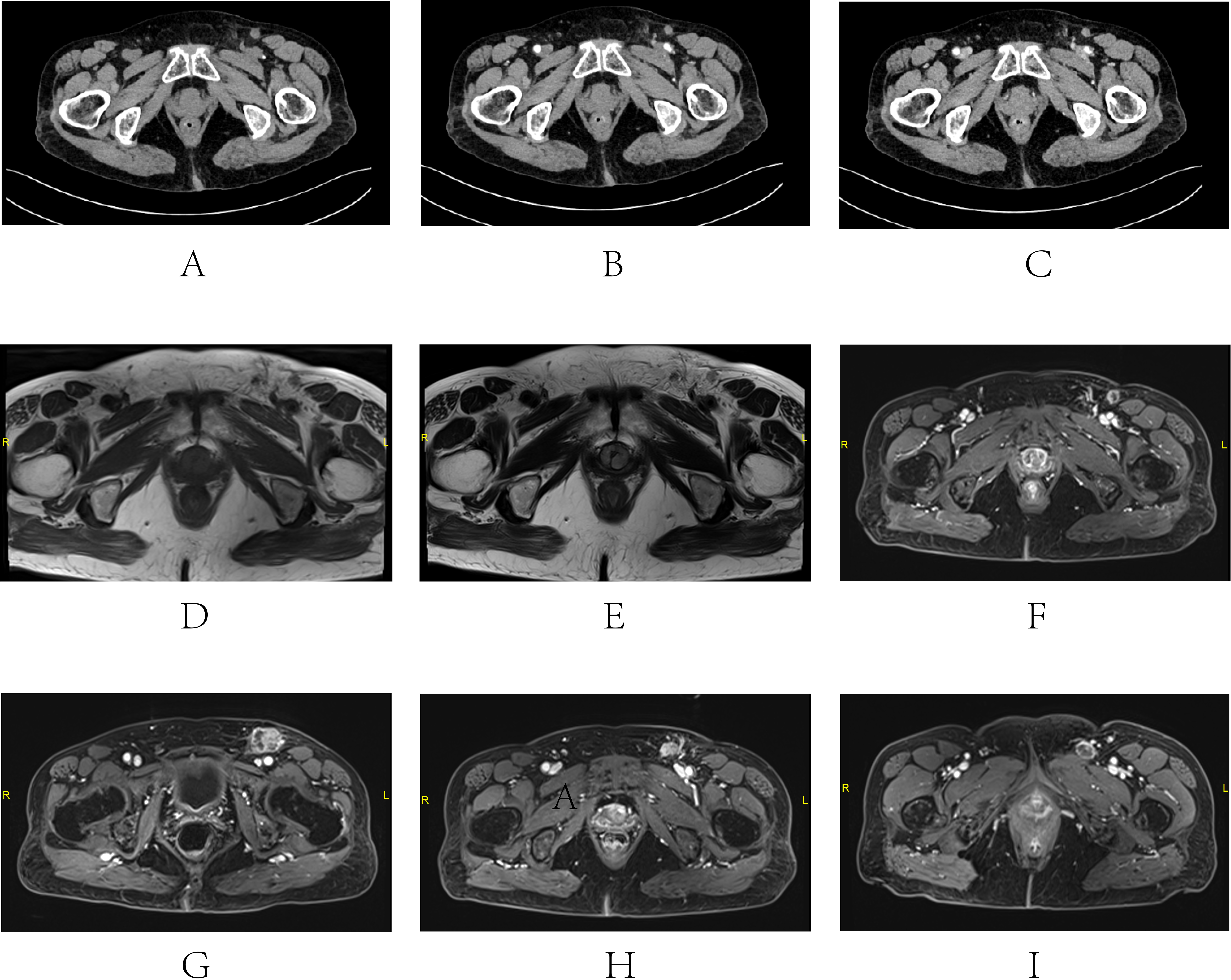

Supplement: Supplementary Figure 2 — Axis scanning of CT and MRI images of Case 1 6 months after chemotherapy and before palliative surgery. CT (A-C) A 2.5∗1.9 cm nodule was found on the urethra, with a plain CT value of 40 HU, and enhancement was visible (70 HU); MRI (D-F) A 2.2*1.8 cm cystic signal was seen with long signal at both T1 and T2 stage with significant enhancement; Multiple enlargements of lymph nodes (G-I) in the left pelvic wall and the inguinal region presented on MRI images. [(A) Plain scan of the mass; (B) Enhanced scan arterial phase; (C) Enhanced scan venous phase; (D) T1 stage of MRI; (E) T2 stage of MRI; (F) Enhanced T1 stage of the mass; (G–I) Enhanced T1 stage of the enlarged lymph nodes]. [file Image2.jpeg]

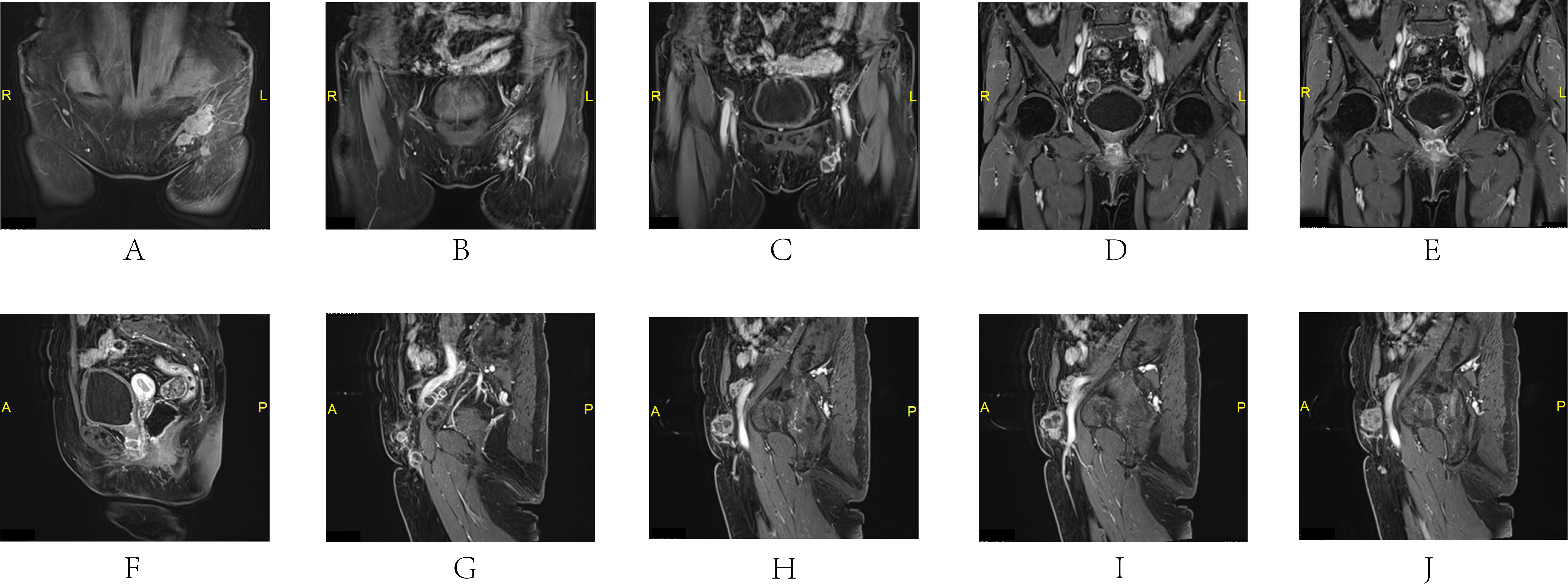

Supplement: Supplementary Figure 3 — Coronal and sagittal images of MRI of Case 1 after chemotherapy recurrence, with a cystic urethral mass and enlarged left pelvic and inguinal lymph nodes. (A–E) Coronal sequences; (F–J) Sagittal sequences. [file Image3.jpeg]

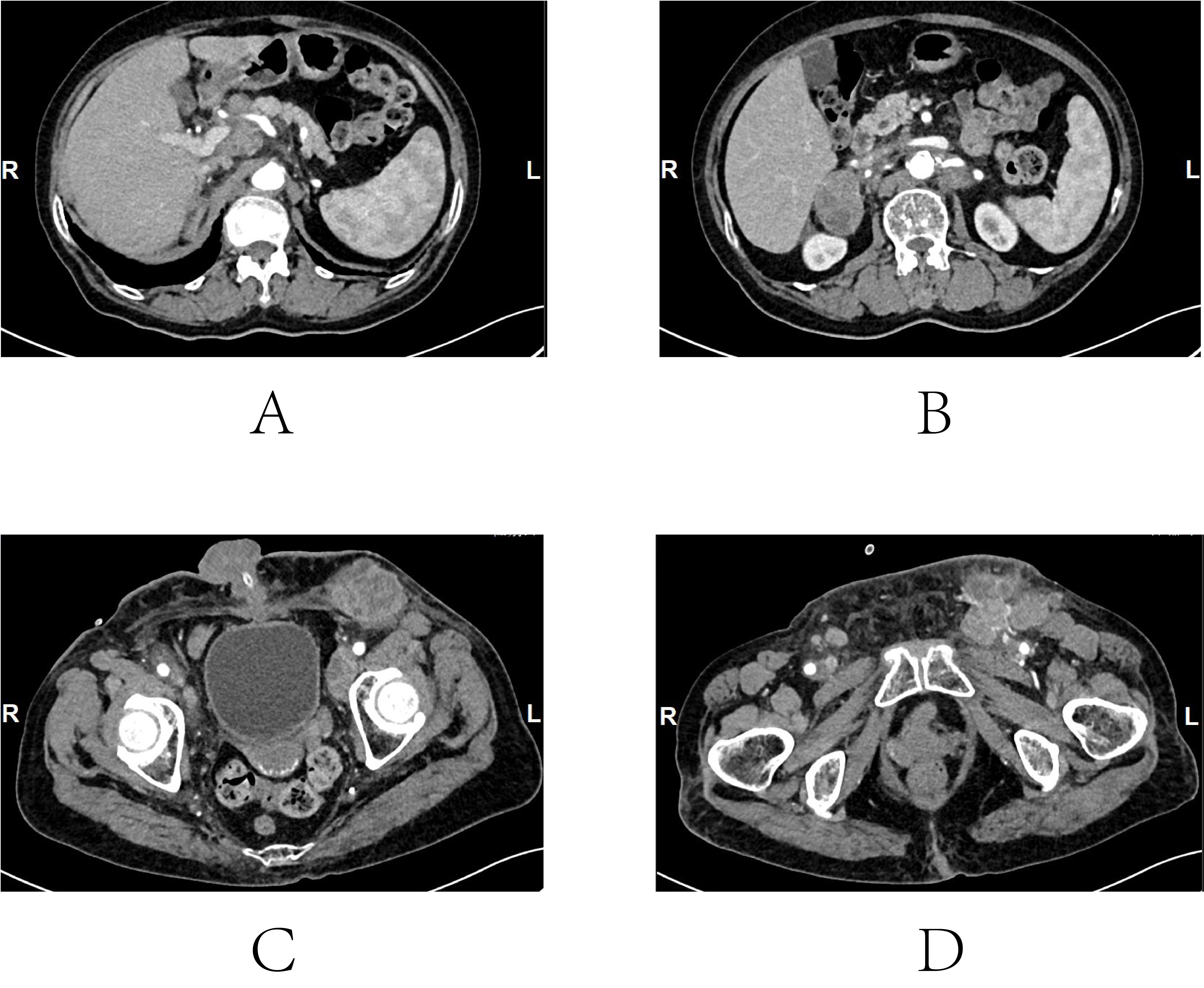

Supplement: Supplementary Figure 4 — Axis CT images of Case 1 (Progression and recurrence 6 months after palliative surgery): (A) Lymph node metastasis at the porta hepatis; (B) Metastatic tumor in the left adrenal area; (C) A mass growing outward from the bladder fistula site and left inguinal lymph node metastasis were presented; (D) Metastases of bilateral inguinal lymph nodes. [file Image4.jpeg]

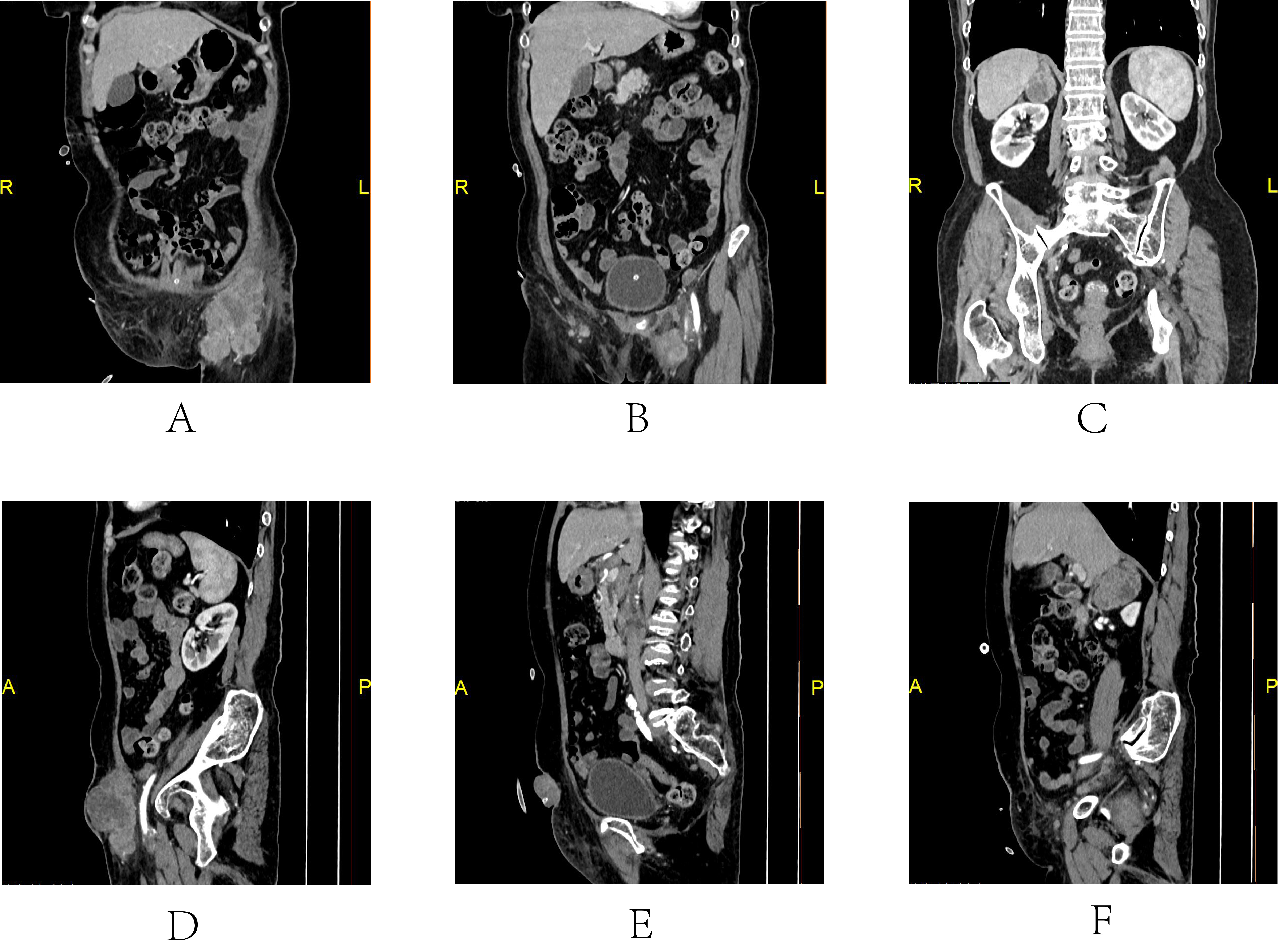

Supplement: Supplementary Figure 5 — Coronal and sagittal images of CT of recurrence after palliative surgery in case 1. (A–C) Coronal sequences; (D–F) Sagittal sequences. [(A) Enlarged and fused left inguinal lymph nodes; (B) Enlarged bilateral inguinal lymph nodes and left pelvic lymph nodes; (C) Metastatic right adrenal tumor; (D) Enlarged and fused left inguinal lymph nodes; (E) A mass growing outward from the bladder fistula site; (F) Metastatic right adrenal tumor]. [file Image5.jpeg]

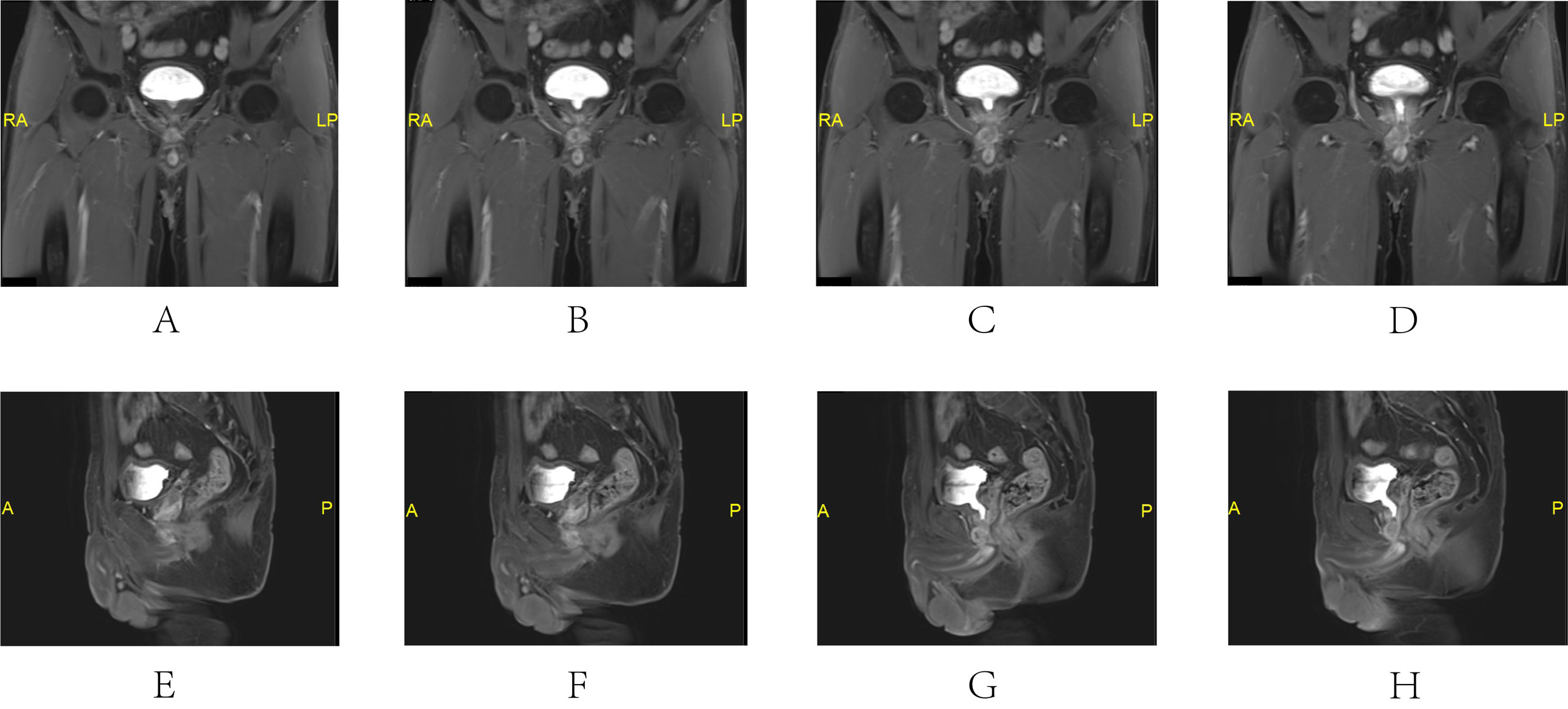

Supplement: Supplementary Figure 6 — Coronal and sagittal images of MRI of Case 2 (with a nodule at the urethral orifice). (A–D) Represented a continuous sequence of coronal images. (E–H) Represented a continuous sequence of sagittal images. [file Image6.jpeg]

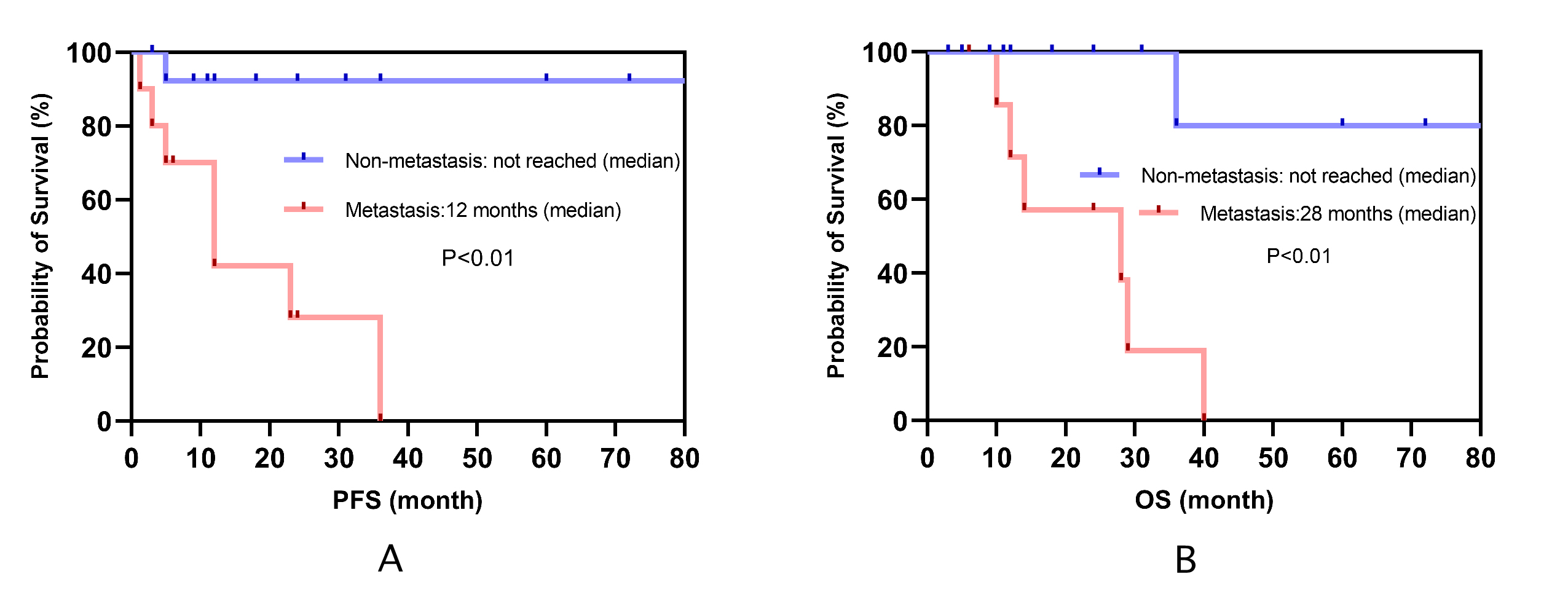

Supplement: Supplementary Figure 7 — Kaplan-Meier Plots of progression-free survival (PFS) and overall survival (OS) of 33 previously reported cases. Consistent with clinical knowledge, patients with detected metastasis prior to treatment have shorter survival times and more common post-treatment progression. It is worth noting that the results of K-M plots can reflect the tendency of the difference in prognosis between these two groups, but may not be accurate enough due to the short follow-up time of most reported cases and the rarity of the disease. (Blue: patients without metastatic sites before primary treatments; Red: patients with detected metastatic sites.) [file Image7.jpeg]
